# I12: the Joint Engineering, Environment and Processing (JEEP) beamline at Diamond Light Source

**DOI:** 10.1107/S1600577515003513

**Published:** 2015-04-08

**Authors:** Michael Drakopoulos, Thomas Connolley, Christina Reinhard, Robert Atwood, Oxana Magdysyuk, Nghia Vo, Michael Hart, Leigh Connor, Bob Humphreys, George Howell, Steve Davies, Tim Hill, Guy Wilkin, Ulrik Pedersen, Andrew Foster, Nicoletta De Maio, Mark Basham, Fajin Yuan, Kaz Wanelik

**Affiliations:** a Diamond Light Source Ltd, Harwell Science and Innovation Campus, Didcot, Oxfordshire OX11 0DE, UK

**Keywords:** high-energy X-rays, tomography, diffraction, SAXS, time-resolved studies

## Abstract

I12 is the Joint Engineering, Environmental and Processing (JEEP) beamline, constructed during Phase II of the Diamond Light Source. I12 is located on a short (5 m) straight section of the Diamond storage ring and uses a 4.2 T superconducting wiggler to provide polychromatic and monochromatic X-rays in the energy range 50–150 keV. The beam energy enables good penetration through large or dense samples, combined with a large beam size (1 mrad horizontally × 0.3 mrad vertically). The beam characteristics permit the study of materials and processes inside environmental chambers without unacceptable attenuation of the beam and without the need to use sample sizes which are atypically small for the process under study. X-ray techniques available to users are radiography, tomography, energy-dispersive diffraction, monochromatic and white-beam two-dimensional diffraction/scattering and small-angle X-ray scattering. Since commencing operations in November 2009, I12 has established a broad user community in materials science and processing, chemical processing, biomedical engineering, civil engineering, environmental science, palaeontology and physics.

## Abstract 2

JEEP is a high-energy (50–150 keV) multi-purpose beamline offering polychromatic and monochromatic modes. It can accommodate large samples and experimental rigs, enabling *in situ* studies using radiography, tomography, energy-dispersive diffraction, monochromatic and white-beam two-dimensional diffraction/scattering and small-angle X-ray scattering.

## 1. Introduction   

Diamond Light Source (DLS) is the United Kingdom’s national synchrotron facility. It is funded by the UK Science and Technology Facilities Council (STFC) and the Wellcome Trust. DLS is being constructed in three phases. The storage ring and seven Phase I beamlines commenced user operation in 2007 (Walker, 2007 ▶; Bartolini, 2007 ▶). A further 15 Phase II beamlines were completed in 2012. Phase III, due for completion in 2017, will bring the total number of beamlines to 33.

I12 is the Joint Engineering, Environmental and Processing (JEEP) beamline, constructed during DLS Phase II. Building commenced in late 2006. User experiments started in October 2009. JEEP meets a strong demand from the UK user community for a high-energy beamline with imaging and diffraction capabilities. High-energy X-rays are essential for penetration through large dense samples of engineering materials such as steel. They also permit the study of materials and processes inside environmental chambers, without un­acceptable attenuation of the beam.

The beamline can operate in polychromatic (‘white beam’) mode or monochromatic mode with a selectable energy between 53 keV and 150 keV. Techniques available to users are: radiography, tomography, energy-dispersive diffraction, monochromatic and white-beam two-dimensional diffraction/scattering and small-angle X-ray scattering (SAXS).

In the beamline design, much attention was given to allow *in situ* studies using the broadest possible range of processing equipment, in terms of size, weight and complexity. Selecting and changing experimental techniques can be done remotely and automatically without the need to manually rearrange beamline instrumentation.

The user community is mainly from the following fields: materials engineering and processing (Korsunsky *et al.*, 2010 ▶; Egan *et al.*, 2012 ▶; Hofmann *et al.*, 2012 ▶; Evans *et al.*, 2012 ▶; Puncreobutr *et al.*, 2013 ▶; Huang *et al.*, 2014 ▶; Kareh *et al.*, 2014 ▶; Davenport *et al.*, 2014 ▶); chemical processing (Williams *et al.*, 2011 ▶; Rowles *et al.*, 2012 ▶; Sedlmaier *et al.*, 2013 ▶); biomedical engineering (Sui *et al.*, 2011 ▶; Zhang *et al.*, 2013 ▶); civil engineering (Bhreasail *et al.*, 2012 ▶); and palaeontology (Baars *et al.*, 2013 ▶).

In this paper we give an overview of the source, optics, detectors and experimental hutch facilities of I12.

## 2. Beamline overview   

**Figure 1:**
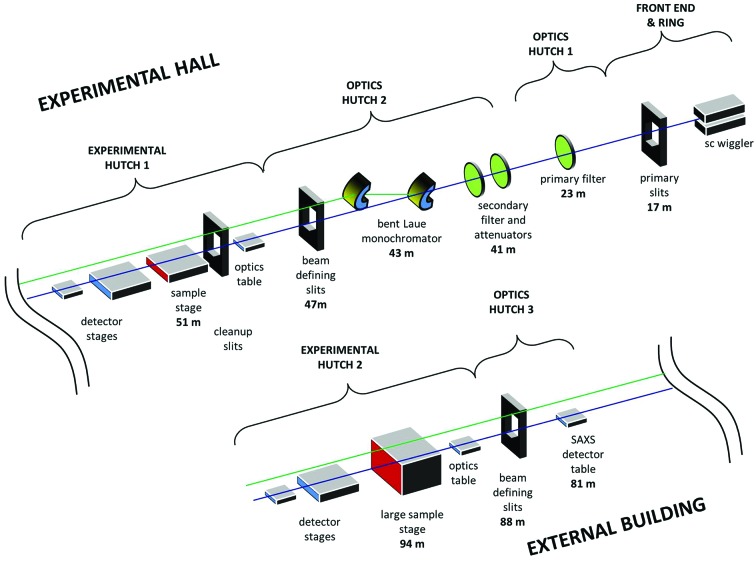
Schematic optical and functional layout of the I12 JEEP beamline.

The beamline schematic is shown in Fig. 1 ▶, with key parameters listed in Table 1 ▶. There are primary slits for beam definition in the front-end section of the beamline. The first optics hutch (OH1), closest to the source, contains the first low-energy X-ray filter, which at the same time is the vacuum window between the front-end section and the beamline. The second optics hutch (OH2) contains a second permanent low-energy filter followed by optional Cu-attenuators of different thickness.

A cryogenically cooled double-crystal bent Laue monochromator and beam-defining secondary slits complete the beamline optics before the first experimental hutch (EH1).

JEEP has an in-line layout with two experimental hutches. EH1 is 51 m from the source and is optimized for experiments on small samples and sample environments. Next is a transfer pipe across the perimeter of the main experimental hall to an external building, where there is a third optics hutch (OH3) with beam-defining slits and a second large experimental hutch (EH2), 94 m from the source. EH2 is used for large samples, experimental apparatus or complicated experiments which require prolonged setup time. The in-line layout means that EH1 can be used for experiments while preparing for another experiment in EH2. The beamline can pass the complete 1 mrad × 0.3 mrad angular fan of either monochromatic or white beam right up to the final beam stop at the end-wall of EH2.

### 2.1. Source   

**Figure 2:**
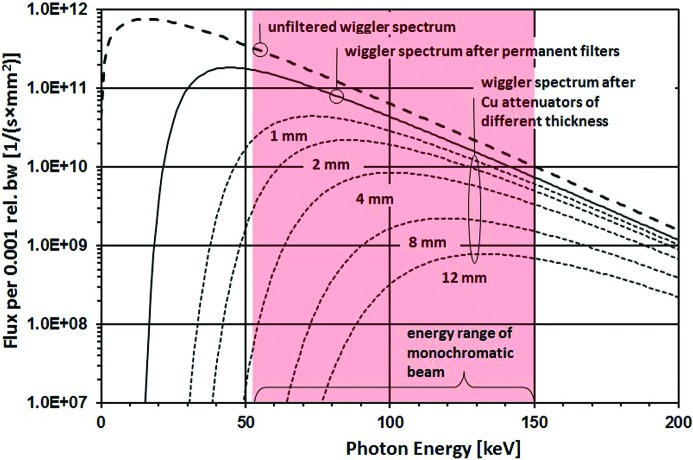
Calculated photon flux at 500 mA in the full 1 mrad × 0.3 mrad fan accepted by the beamline at a distance of 50 m from the source (EH1). The effect of fixed filters and selectable white-beam attenuation is shown.

Diamond is a 3 GeV synchrotron source. At the time of writing it operates at 300 mA, in top-up mode, with a design target of 500 mA. The I12 insertion device is a 4.2 T superconducting wiggler with 21 full-field periods of 48 mm period length (Budker Institute for Nuclear Physics, Novosibirsk, Russia). The resulting deflection parameter *K* is 18.8 and the critical energy is 25 keV, with a total power of 56 kW at a storage ring current of 500 mA. A fixed front-end aperture restricts the maximum angular acceptance of the beam to 1 mrad horizontally and 0.3 mrad vertically, so the maximum power entering the beamline is approximately 9 kW at 500 mA. Fig. 2 ▶ shows the flux in the 1 mrad × 0.3 mrad fan at 300 mA, calculated using *XOP* (Sanchez del Rio & Dejus, 2004 ▶). With the critical energy of the wiggler at 25 keV, about two-thirds of the total power is in photons below 50 keV and thus below the operating energy range of the beamline.

### 2.2. Beam conditioning optics and heatload management   

The front-end contains a set of primary slits which are used to reduce the beam size entering the beamline if necessary. The maximum beam size in EH1 is 50 mm × 15 mm and 94 mm × 28 mm in EH2, corresponding to a maximum angular fan width of 1 mrad × 0.3 mrad, at sample position.

The high radiation intensity emitted from the wiggler requires careful design of heatload management (Patterson *et al.*, 2005 ▶). Two fixed filters are permanently inserted into the beam path with the aim to reduce the power at energies below 50 keV as much as possible. This is achieved by subsequent beam hardening at the two filter locations.

The first filter at 23 m from the source consists of two 1.1 mm-thick diamond disks of fluorescence-grade chemical vapour deposited (CVD) diamond (Element Six). The two disks are water cooled and mounted in sequence. The average power before the filter is 58 W mm^−2^. The diamond disks reduce the total power from 9 kW to 6.2 kW. Both disks are diffusion bonded to a copper body *via* a molybdenum interface. The first disk acts also as the vacuum window between the beamline and the storage ring. The second disk, identical in design, is located 0.2 m downstream of the first. It is a backup window to protect the storage ring vacuum in case of first disk failure. In addition, the first disk provides an online beam monitor *via* an X-ray induced visible-light luminescence image, which is recorded with a CCD camera positioned outside a sapphire viewport.

The second filter at 41 m from the source is a 4 mm-thick CVD silicon carbide (SiC) disk which is diffusion bonded to a water-cooled copper body *via* a molybdenum interface. The average power density before this filter is 12 W mm^−2^. The second filter reduces the total power from 6.2 kW to 2.6 kW. All subsequent optical elements such as optional attenuators, the first monochromator crystal, slit blades and beamline windows are subject to this level of incident power, at maximum beam size. The cumulative effect of the beamline filters on the incident spectrum is shown in Fig. 2 ▶. At a distance of 50 m, the remaining average white-beam power is 3.5 W mm^−2^. The power in the most intense on-axis central square millimetre is 7 W.

Besides the thermal properties, the choice of filter material takes into account beam hardening characteristics, vacuum compatibility and, very importantly, the avoidance of inhomogeneous amplitude or phase modulation of the transmitted X-rays.

After the SiC filter are two in-vacuum translators with different thicknesses of copper filter (1, 2, 4 and 8 mm). The translators can be set to vary the amount of additional filtration (up to 12 mm). These filters provide additional power management and spectrum hardening, for example when commissioning equipment, or for filtered white-beam experiments such as high-speed imaging.

### 2.3. Monochromator   

The I12 monochromator is a double-crystal bent Laue geometry instrument using cryogenically cooled Si single crystals. The device is designed to provide large high-energy beams for full-field imaging, at high intensity and moderate spectral resolution, but also monochromatic beams with high spectral resolution for diffraction. An engineering and optical design study for the monochromator was undertaken to check the validity of the optical and thermal engineering concept (Sutter *et al.*, 2008*a* ▶,*b* ▶).

At high photon energies, silicon is transparent to X-rays, making the Laue geometry feasible (Suortti & Schulze, 1995 ▶; Shastri *et al.*, 2002 ▶). The I12 monochromator uses two silicon crystals of almost identical design and optical layout. The optically active part of the crystals is a 4 mm-thick rectangular slab with both surfaces polished to topographic quality. The Si (111) reflection is chosen over the complete energy range from 53 keV to 150 keV. The diffraction vectors are oriented vertically in a (+,−) setting, producing a monochromatic beam with a vertical offset of 50 mm above the incident white beam. The (111) lattice planes and the surface normal include an angle of 44°, for both crystals. The normal vectors of the crystal surface directed towards the source point upwards. The X-rays impinge onto the first crystal surface inside the angle between surface normal and the lattice planes, and outside this angle on the second crystal.

Both crystals are cylindrically bent in the vertical, meridional, plane with the concave surface directed towards the source. The bending can be changed from unbent to a smallest bending radius of about 35 m. In the chosen diffraction geometry, the monochromatic focusing effects from both bent crystals compensate each other almost completely. Thus the initial vertical divergence of the X-ray beam is not altered by the monochromator, in a first approximation.

Both crystals are cooled. The first crystal, at a distance of 43 m from the source, absorbs 1 kW in a volume measuring 43 mm × 18 mm × 4 mm (horizontal × vertical × thickness). To reduce thermal strains at such heat load, the first crystal is cryogenically cooled *via* Cu-absorbers which are clamped onto the peripheral parts of the crystal. The second crystal is connected to a cryogenically cooled runner *via* a Cu-braid. Although the thermal conductivity of the braid is poor, the second crystal can be kept reliably at temperatures below 250 K which at high energies is sufficient to reduce the dispersive nature of the second crystal below practical importance.

**Figure 3:**
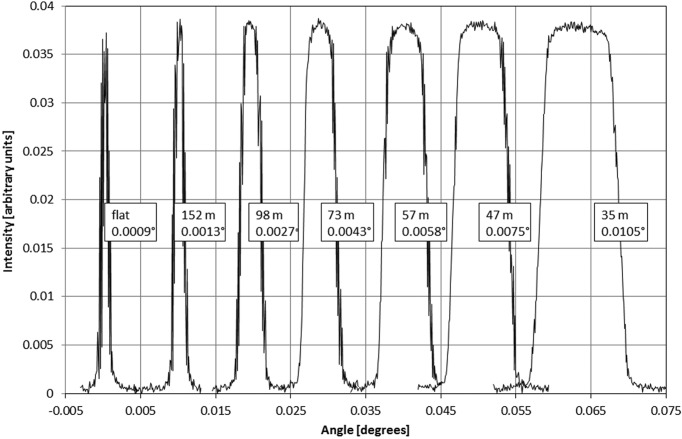
Rocking curves at 50 keV of one bent Laue Si (111) crystal at different bending radii measured with a Si (111) Bragg crystal and a pencil beam. Width (FWHM) and intensity are shown for different crystal bending radii from flat to 35 m.

The spectral bandwidth is controlled through the crystal bending (Fig. 3 ▶). For imaging, a broad bandwidth can be tolerated to maximize the photon flux at the sample. For diffraction, where better energy resolution is desirable, the bandwidth can be narrowed by reducing the crystal bending.

### 2.4. Experimental hutches   

Two experimental hutches are available. EH1 (sample at 51 m from the source) is located inside the experimental hall. EH2 (sample at 94 m from the source) is housed in an external building outside the hall. Both sample positions are centred on the middle of the beam. The two experimental stations have a similar layout. Both are optimized to allow switching between imaging and diffraction techniques on the same sample without manual intervention.

The underlying design concept for both experimental hutches is to enable *in situ* studies with the greatest possible variety of sample environments, without having to extensively adapt equipment for use with synchrotron X-rays. Ideally, users should be able to bring laboratory instrumentation and use it in the I12 X-ray beam with minimal modifications. The main characteristics of high-energy X-rays support this concept. First of all, the good penetration reduces the requirement for thin samples or sample cells. Secondly, scattering angles are small, thus scattered X-rays are confined within a narrow cone. Diffraction and scattering can be recorded at distances of several hundreds of millimetres downstream of the sample, allowing more space between sample and detector for a sample rig or chamber. Finally, in-line phase-contrast *via* propagation develops at distances only above several 10 mm behind the sample, which allows recording pure absorption contrast at a comfortable sample–detector distance if needed.

The continuous spectrum from the wiggler can be used for energy-dispersive diffraction. With collimation, a three-dimensional gauge volume can be produced, enabling isolation of the sample signal from unwanted contributions from sample containers or other material in the beam path.

All the factors mentioned above enable the provision of ample space around the sample position. For this reason, the I12 sample stages are designed to be sturdy and capable of supporting heavy weights and large objects.

Both hutches have a table before the sample stage for additional equipment and optics, and a large detector table with motions parallel and perpendicular to the incoming X-ray beam after the sample stage. Up to three detectors can be mounted simultaneously. They can be moved remotely during an experiment, for example to switch between imaging and diffraction. Sample-to-detector distances can be varied from ∼0.6 m to 2.6 m for both imaging and diffraction detectors. In-line phase contrast propagation distance and the range of momentum transfer in diffraction can be thus adapted to various needs.

At the end of EH1 and EH2, at a distance of 5 m and 7 m to the sample stage, respectively, a second detector table with 2 m horizontal travel perpendicular to the X-ray beam allows the recording of diffraction signals with high angular resolution.

EH1 is configured for experiments with relatively small samples and sample environments when compared with EH2. A core feature is a multi-purpose sample stage (Huber Diffraktionstechnik GmbH) which can be configured to suit different sizes of experimental equipment. There is a high-precision air bearing rotation stage for tomography, which can rotate at up to 10 Hz. The sample stage can carry up to 50 kg with the tomography stage installed and up to 200 kg with the tomography stage removed.

EH2 differs from EH1 in that it offers additional design features for the setup and study of large samples and complex sample environments. Most notably, the sample stage (Max Voggenreiter GmbH) in EH2 can support masses up to 2000 kg with a horizontal and vertical motion range of 1 m and a continuous rotation around a vertical axis. EH2 is equipped with 1.9 m-wide by 3.0 m-high entrance doors, a 5 tonne capacity overhead crane, various conduits for gases and liquids and a hot fume extract. The external building has its own loading bay for delivery of large items of equipment or samples. Before an experiment, complex instrumentation can be assembled and tested in EH2, whilst beamline operation continues in EH1.

Finally, taking advantage of the long X-ray transfer tube to the external building, a facility for high-energy SAXS is included in the beamline capabilities. This was achieved by enlarging the transfer pipe to a diameter of 400 mm, and constructing a detector station in Optics Hutch 3, at a distance of 30 m from the sample position in EH1.

### 2.5. Detectors and related techniques   

#### 2.5.1. X-ray imaging cameras   

**Figure 4:**
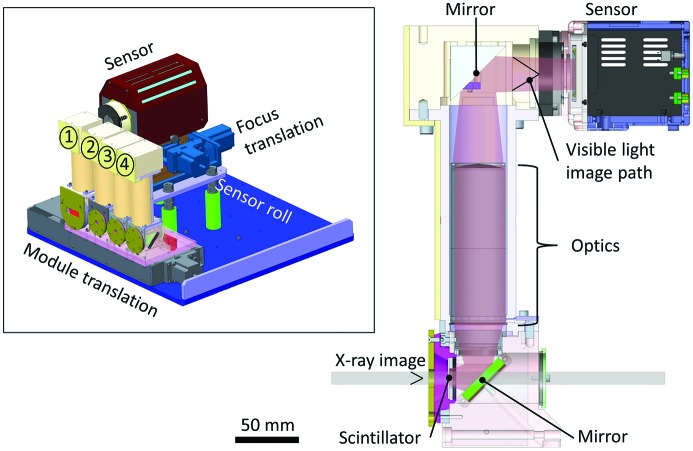
Schematic design of the I12 imaging camera. X-rays enter an optics module (bottom left). A visible-light image is generated in the scintillator and imaged onto a commercial sensor (top right). The visible-light path is folded twice. The inset shows the modular design with four optical modules for different magnification on a linear translation and the sensor on a focus translation and a roll stage.

The main I12 imaging camera was designed in-house. It has a modular construction, aiming to achieve high robustness with a minimum degree of adjustment required for magnification changes (Fig. 4 ▶).

Four optics modules with different fields of view are mounted side by side on a linear translation stage. Each module consists of an imaging scintillator, visible-light mirrors and optics. For optimum spatial resolution and light efficiency, the thicknesses of the scintillator are matched to the depth of focus of the optical system (Koch *et al.*, 1998 ▶) for each module. Each optics module creates a real image of the scintillator at a fixed distance behind the module. That image is recorded by a commercial CCD or CMOS sensor. The field of view is changed by translating the according optics module before the sensor. Fields of view, magnification and pixel resolutions are listed in Table 2 ▶.

No mechanical or optical adjustments are necessary for any of the modules, because the manufacturing machining was sufficiently accurate to achieve the required tolerances of parallelism, straightness and distances. Focusing of the scintillator is done in image space by moving the sensor along the optical axis. As the lateral magnification is proportional to the square of the object magnification, focusing is easier when performed in image space, and there is no need for high-resolution motion mechanics at the scintillator location.

The light from the scintillator is reflected upwards by a 45° mirror. Custom-made optics image the scintillator onto the sensor plane at the desired magnification. Before reaching the sensor, the light is reflected by a second 45° mirror into the horizontal direction again. The double-mirror design keeps the optics and camera sensor out of the direct X-ray beam path, and allows easy translation or removal of the optics modules. All four optics have identical nominal object–image distances. The optical glass used is radiation-resistant, in order to decrease long-term radiation damage caused by high-energy elastic and Compton scattering which originates from sample, scintillator and first mirror.

Currently two interchangeable image sensors are available. High-resolution imaging is performed using a PCO.edge monochrome camera (PCO AG), with a 2560 × 2160 pixel CMOS sensor and a maximum acquisition rate of 100 frames s^−1^. Images are continuously acquired and saved on central storage at maximum frame rate. For high-speed imaging, an 800 × 600 pixel Phantom 7.3 CMOS camera is used (Vision Research). The camera is capable of up to 6600 full frames per second, more if a reduced region of interest is selected. During acquisition, images are stored in a 16 Gigabyte capacity on-board memory. The images are then downloaded to central file storage over an Ethernet connection. The sensors can be rotated around the optical axis by a few degrees. This enables adjustment of the tomography rotation axis to be orthogonal to the sensor rows in cases when a tilt stage under the tomography rotation axis is not available.

The scintillators are mounted in magnetic holders which engage with magnets on the optic module body, enabling quick simple exchange of scintillators. The lateral position is defined by precisely machined datum planes. Using this system, the scintillator thickness can be adapted to the resolution of the sensor or to increase efficiency at the expense of resolution. The main scintillator materials used are single crystals of cadmium tungstate, cerium-doped lutetium aluminium garnet and terbium-doped gadolinium gallium garnet.

#### 2.5.2. Flat panel detector   

Recent developments in digital medical imaging technology made large, flat, two-dimensional detectors suitable for beamline use commercially available (Daniels & Drakopoulos, 2009 ▶). A Pixium RF-4343 (Thales) flat panel detector was selected for two-dimensional powder diffraction, Laue-diffraction, total scattering and SAXS experiments. It has 2880 × 2881 pixels, each 148 µm by 148 µm, in a total active area of 430 mm × 430 mm. X-ray to visible light conversion takes place in a columnar crystalline CsI scintillator array. The visible light is detected by an amorphous silicon photodiode array. The processed signal is written to a two-dimensional tiff-file with 16-bit depth. The direct beam is blocked by a cylindrical tungsten beamstop, which is supported using a low-absorbance carbon fibre arm to minimize shadowing of diffraction patterns on the detector. Automated routines for calculating the monochromatic beam energy, sample-to-detector distance, detector tilt and beam centre position have been developed. The calculations use a number of diffraction patterns from a known standard (*e.g.* NIST 674b cerium oxide) captured over a range of sample-to-detector distances (Hart *et al.*, 2013 ▶).

As well as monochromatic powder diffraction, the flat panel detector has been used for high-energy transmission Laue diffraction with white beam (Hofmann *et al.*, 2012 ▶).

#### 2.5.3. Energy-dispersive detector   

Energy-dispersive X-ray diffraction (EDXD) is an established technique for the retrieval of structural information from samples inside equipment with confined geometries, such as environmental cells, furnaces or pressure cells. To optimize the beam time utilization of the energy-dispersive technique, a multi-element detector was designed, with 23 high-purity Ge detector crystals arranged in a semi-annular array with a diameter of 350 mm. The azimuthal angles cover a range from 0° to 180°, in steps of 8.18°. Each detector crystal is cylindrical in shape with a diameter of 10 mm and a depth of 14 mm. The detector was manufactured by Canberra, Olen, Belgium, to a conceptual design provided by the I12 beamline. Detector readout is performed by an XMAP digital acquisition system (XIA Inc.).

The semi-annular array of detector elements makes up 11 orthogonal pairs in total (plus one additional element), allowing the simultaneous measurement of 11 sets of orthogonal *q*-vector components. This geometry is intended to improve the determination of strain tensors and also permits the measurement of texture. The multi-element design also increases the detected total solid angle which is an advantage for the acquisition of patterns without azimuthal signature, for instance in chemical processing.

At a typical sample–detector distance of 2 m the diffraction angle 2θ is 5°, which at the nominal useable energy range from 50 keV to 150 keV gives access to a momentum transfer *q* from 2.2 Å^−1^ to 6.5 Å^−1^.

**Figure 5:**
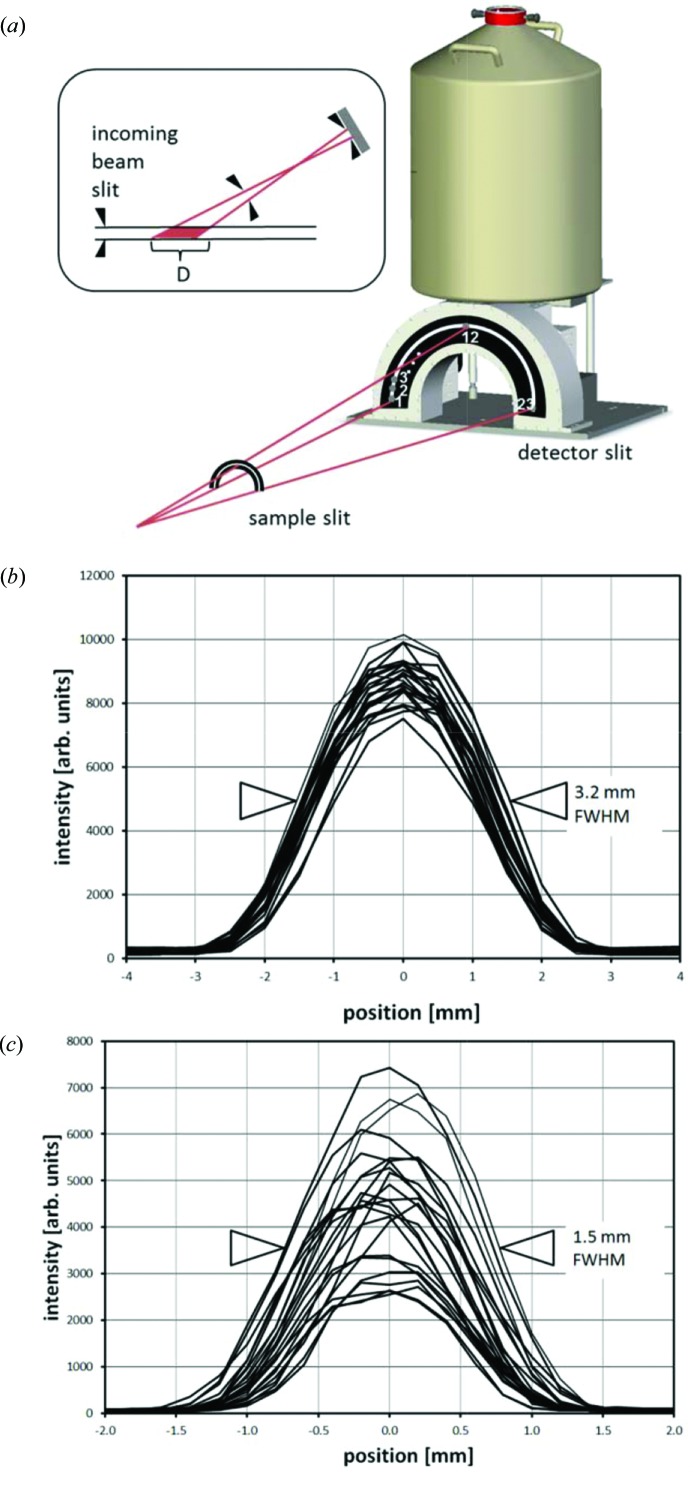
The EDXD system. (*a*) Geometry of the detector, detector slits and sample slits showing the semi-annular arrangement of 23 independent Ge crystals. Inset: geometry of gauge-volume dimension *D* along incoming beam direction. Measured length *D* and relative position of gauge-volume of all 23 elements at 50 µm × 50 µm incoming beam size for long collimator (*b*) and short collimator (*c*).

The complete EDXD set-up has a semi-annular slit array mounted on the entry face of the detector (detector slit) and a second semi-annular slit with fixed gap not far behind the sample (sample slit). Both these slits form a collimation for the diffracted beam. Together with the confinement of the incident beam through the two orthogonal pairs of entrance slits a three-dimensional gauge volume is defined. The length of this volume along the beam is given by the entrance slits and the geometrical parameters of the collimation (sample, and detector slit gap and the distances between them and the sample) (Fig. 5*a* ▶). The collimation parameters also define the residual divergence of the diffracted beam around the nominal take-off angle. The parameters are balanced in a way that the residual divergence is small compared with the intrinsic energy resolution of the detector, in terms of momentum transfer *q*. The shallow diffraction angles elongate the gauge volume along the beam direction (Rowles, 2011 ▶).

A three-dimensional gauge volume is extremely useful for strain scanning or when diffraction from material surrounding the sample needs to be discriminated at the detector. To permit some flexibility in gauge volume length and sample to sample-slit distance, different sample slits are available. The smallest gauge-volume length of 1.5 mm (full width at half-maximum) can be achieved with a sample slit located 100 mm behind the sample (Fig. 5*c* ▶). Here, the centre positions of the 23 individual gauge-volumes overlap within a tolerance of less than 0.3 mm. The uneven intensity over the different elements reflects the mechanical tolerances of the conical slit opening which is nominally 70 µm, cut into an 8 mm-thick tungsten plate. A larger gauge volume with a length of 3.2 mm (full width at half-maximum) can be provided with a different sample slit at a distance of 515 mm behind the sample (Fig. 5*b* ▶).

#### 2.5.4. Sample environments   

The main objective of JEEP is facilitating *in situ* experiments on samples and systems where high-energy X-rays are needed, either for good penetration through dense material or for high coverage of the reciprocal space in a forward diffracting geometry. The main applications therefore originate from materials and engineering science, and chemical processing; however, a significant number of applications come from physics, medical research and geoscience.

Since the first days of operation in October 2009, the user community has brought state-of-the-art sample environments to the beamline. At present, about 85% of experiments use equipment and sample environments for *in situ* studies. The range of user sample equipment brought to date is vast, and the complexity can be high. Most of the equipment is tailored to carry out specific sample operations and processes for the scientific problem under study. The equipment is operated at the users’ home laboratories, as well as at JEEP. Prominent examples include: a tomography rig for controlled heating, cooling and deformation of samples (Puncreobutr *et al.*, 2012*a* ▶,*b* ▶); a gas gun for shock physics experiments (Eakins & Chapman, 2014 ▶); molten salt electrowinning apparatus (Styles *et al.*, 2012 ▶); aluminium casting apparatus (Drezet *et al.*, 2014 ▶); welding rigs; an internal combustion engine (Baimpas *et al.*, 2013 ▶); chemical processing equipment (Lester *et al.*, 2006 ▶); hydro-thermal reactors (Moorhouse *et al.*, 2012 ▶); a magma chamber (Pankhurst *et al.*, 2014 ▶); a custom furnace for studying solid oxide fuel cells (Robinson *et al.*, 2014 ▶) and various mechanical test rigs (Evans *et al.*, 2012 ▶; Horne *et al.*, 2013 ▶; Mostafavi *et al.*, 2013 ▶; Gussone *et al.*, 2014 ▶).

Due to the wide range and sophistication of user requirements, it would be difficult and costly for the beamline to provide all the equipment required for *in situ* experiments. Instead, the beamline offers some generic apparatus, including furnaces, a cryo-streamer (Oxford Instruments) and a 100 kN servo-hydraulic mechanical test-rig (Instron, UK).

#### 2.5.5. Control system   

Like the rest of Diamond, beamline I12 uses the EPICS distributed control system for low-level control (APS-ANL, 2014 ▶). The user interface for experiments and data acquisition is the Generic Data Acquisition system (GDA, 2014 ▶), an open source software suite originally developed at the Daresbury Synchrotron Radiation Source and adopted by Diamond Light Source. For customized experimental control, Jython scripts can be written and executed within the GDA software.

## 3. Ancillary facilities   

### 3.1. Support laboratories   

There are two general-purpose support laboratories adjacent to the beamline, equipped with optical microscopes, a fume cupboard and basic chemical laboratory equipment. Diamond Light Source users may request access to the facility’s central chemistry and biological science laboratories. Equipment in the support laboratories of other beamlines may also be available to users on request.

### 3.2. Computing facilities   

All data collected on the beamline is stored centrally on a high-performance GPFS file system and can be accessed remotely by users after their experiment is complete.

Given the high demand for imaging and tomography, significant resources have been deployed for image processing and tomographic reconstruction. For fast reconstruction, there is a central computing cluster based on Nvidia® Tesla Graphical Processing Units (GPUs). Tomography reconstruction uses a filtered back projection algorithm with ring artefact suppression, optimized for parallel processing on GPUs and developed in collaboration with the University of Manchester (Titarenko *et al.*, 2010 ▶, 2011 ▶; Kyrieleis *et al.*, 2011 ▶). Single-distance phase retrieval based on the method proposed by Paganin *et al.* (2002 ▶) and implemented in the software *ANKAphase* (Weitkamp *et al.*, 2011 ▶) is available. Recently, new algorithms for improved determination of the tomography rotation centre (Vo *et al.*, 2014 ▶) and a new method for phase retrieval from multi-distance tomography datasets has been developed (Vo *et al.*, 2012 ▶), using the Gerchberg–Saxton algorithm (Gerchberg & Saxton, 1972 ▶).

The beamline has a data analysis room where users can process and visualize data while they are on site, or during a separate visit after the experiment. Several commercial and free packages are available, including *AVIZO* (FEI Visualization Sciences Group) for visualization and rendering of three-dimensional computed tomography data. For the calibration, reduction and analysis of diffraction data from either two-dimensional powder diffraction or EDXD, Diamond has been developing a powerful module within the open source ‘DAWN’ data analysis workbench (Basham *et al.*, 2015 ▶). Calibration of monochromatic two-dimensional powder diffraction data is performed using algorithms developed at DLS (Hart *et al.*, 2013 ▶). For full Rietveld pattern refinement of diffraction data, the *TOPAS* software package is available (Bruker, 2009 ▶).

Diamond Light Source is continuing to develop the GDA and DAWN software suites to provide a complete workflow for data collection, reduction, analysis and visualization. For I12 in particular, workflows and graphical user interfaces for tomography reconstruction and powder diffraction data reduction are available and will continue to be updated and improved.

## 4. Facility access   

DLS provides two general user access methods for beamline I12, Direct and Programme Mode. In Direct Mode, beam time allocation is in two periods of six months starting October and March. Users can submit proposals twice a year with deadline dates usually on 1 April and 1 October. Applications are peer reviewed on scientific merit by an independent panel of scientists. Typically, users on I12 receive between two and five days of beam time per application. Programme Mode provides access over a period of up to two years, with two calls per year at the same time as Direct Mode.

## 5. Experimental highlights   

There follows some selected examples of published research, showing the versatility of the JEEP beamline and the diversity of experiments performed.

### 5.1. Engineering   

An early experiment in EH2 was a feasibility study of high-speed imaging and strain measurement in an operating single-cylinder four-sroke internal combustion engine running at ∼2000 r.p.m. High-speed radiography was used to observe the motion of valves, piston and connecting rod. Stroboscopic energy-dispersive diffraction measurements on the connecting rod revealed a compressive strain of −630 microstrain at its position close to the top dead centre after ignition (Baimpas *et al.*, 2013 ▶).

### 5.2. Material science   

JEEP has regularly performed high-speed *in situ* tomography experiments on the deformation of semi-solid systems. Such systems occur in natural environments like magmas or soil, and also in industrial processes like the casting of alloys. A particular highlight was the direct measurement of discrete grain response during compression of a semi-solid Al–Cu alloy (Kareh *et al.*, 2014 ▶). The stress–strain response was due to the shear-induced dilation of discrete rearranging grains, leading to the counter-intuitive result that compression can open internal pores and draw the free surface into the liquid, resulting in cracking.

Another *in situ* experiment, using diffraction, was a total scattering study of the deformation mechanisms in a Zr–Ti-based bulk metallic glass composite, investigating the correlation between local atomic re-arrangement and shear band formation in the presence of a stress concentration (Huang *et al.*, 2014 ▶).

### 5.3. Chemical processing   

A new superconductor, made by an intercalation reaction, was synthesized on JEEP. The reaction was monitored *in situ* using time-resolved 2D-diffraction at 80 keV (Sedlmaier *et al.*, 2013 ▶). The resulting product was an ammonia-rich intercalate of FeSe with a critical temperature *T*_c_ of 39 K.

### 5.4. Palaeontology, biomedical imaging   

High-resolution tomography has enabled successful non-invasive examination of a partially silicified fossil in a calcareous rock matrix. The internal structures of the fossil were revealed in sufficient detail to unambiguously identify it as the earliest rugose coral known to date (Baars *et al.*, 2013 ▶).

Biomechanics plays an important role in glaucoma. Elevated eye pressure is the main risk factor for developing this disease. It is believed that pressure-induced deformation of the connective tissues of the back of the eye causes degeneration of the neural cells that transmit the visual information. The anatomical complexity, small size and relative inaccessibility of the involved bio-mechanics make phase-contrast imaging an ideal method to measure the pressure-induced deformation of this tissue at high resolution. Human donor eyes were attached to a pressure chamber and maintained inflated during tomographic data-acquisition. Pressure-induced deformation of retina supporting tissue was observed and quantitatively analysed (Coudrillier *et al.*, 2015 ▶).

### 5.5. Instrumentation   

A novel pixelated CdTe detector (Jacques *et al.*, 2013 ▶) was used in a pinhole imaging setup to record dark field powder diffraction images of heterogeneous samples *via* the energy-dispersive method. One image consists of a square array of 80 × 80 individual diffraction patterns at a spatial pixel resolution of 100 µm × 100 µm at the object (Egan *et al.*, 2014 ▶).

## 6. Planned upgrades   

### 6.1. X-ray optics   

With the target of storage ring operation at 500 mA, work is in progress on a new secondary filter. The current CVD SiC filter is vulnerable to cracking due to thermally induced stress, exacerbated by residual stresses remaining from the diffusion bonding process. The new design should reduce thermally induced stresses and make filter replacement easier if it fails.

There is an ongoing modification programme for the monochromator, mainly to improve mechanical reliability and eliminate components such as optical encoders which are sensitive to beam damage. It should be noted that the X-ray optical performance of the device has been excellent since the first days of operation in 2009, and only one experiment has had to be cancelled due to a problem that could not be fixed in time.

A setup to improve the *q*-resolution in high-energy SAXS is in the early stages of testing. Using compound refractive lenses, the extended beam at the sample is focused onto the detector. Due to the high quality of the lenses used, the focused pattern retains the high brilliance of the source (Drakopoulos *et al.*, 2005 ▶).

### 6.2. Tomography   

A new large field of view (LFV) camera is currently being commissioned to cover the large imaging beam in EH2. The camera has a field of view of 104 mm × 46 mm. It uses two scientific grade CMOS sensors (PCO.edge) with separate optics. With it, the scintillator is imaged in two separate horizontally adjacent parts with some minor overlap in the centre. The final image has a size of 4810 × 2160 pixels at a pixel size of 21.5 µm. The light paths are folded horizontally and extensive tungsten shielding is applied to virtually eliminate any scattered X-rays from striking the camera sensors or optics.

For high-speed tomography in EH1, a dedicated white-beam imaging module is being manufactured, and the EH1 tomography stage is being upgraded to a rotation speed of 10 Hz.

### 6.3. Diffraction detectors   

A high-speed two-dimensional detector for diffraction, called L-AXIS, has been tested on the beamline and is ready for commissioning. The device was developed and produced by the STFC Technology Department of the Rutherford–Appleton Laboratories in Harwell. Its detecting element consists of a CMOS monolithic active pixel sensor coupled to a 1.5 mm-thick columnar CsI:Tl scintillator. The array-size is 450 × 450 pixels at a pixel size of 120 µm. At full resolution, 450 frames s^−1^ can be continuously acquired and saved. Higher speeds are possible with a binned sensor.

## 7. Conclusion   

This paper has described the design, layout and operational capabilities of the I12 JEEP beamline at Diamond Light Source. The beamline is unique for a 3 GeV source in offering a large white or monochromatic X-ray beam (up to 100 mm × 30 mm) for imaging, and also small beams for energy-dispersive and angular-dispersive diffraction and scattering. The two experimental hutches, flexible layout and choice of detectors make JEEP a versatile instrument with a broad user community. The versatility of the beamline is illustrated by the variety of experimental work published since the beamline started operations in November 2009. Several optics and instrumentation developments are under way to further improve beamline performance and offer new opportunities to users.

## Figures and Tables

**Table 1:** Key parameters of the I12 JEEP beamline

Source	Superconducting wiggler, 4.2T, 48mm periodicity, 21full-field periods
Beam acceptance	1mrad (H) 0.3mrad (V)
Working energy range	53150keV
Beam modes	White and monochromatic
Monochromator	Si (111) cryo-cooled double bent Laue
Bandwidth	2 10^3^ to 2 10^4^, adjustable
Maximum beam size (EH1)	50mm (H) 15mm (V)
Maximum beam size (EH2)	94mm (H) 28mm (V)
Photon flux (EH1, 53keV at 300mA ring current)	1.8 10^11^ photons s^1^ mm^2^ (0.1% bandwidth)^1^

**Table 2:** X-ray imaging camera optics summary

Module number	Magnification	Field of view High resolution (PCO.Edge) (mm)	Pixel scale High resolution (PCO.Edge) (m pixel^1^)	Field of view High speed (Phantom 7.3) (mm)	Pixel scale High speed (Phantom 7.3) (m pixel^1^)
1	0.346	48.8 40.5†	19.1	50.9 38.2†	63.6
2	0.82	20.3 17.1†	7.9	21.5 16.1†	26.8
3	2	8.3 7.0	3.2	8.8 6.6	11.0
4	5	3.3 2.8	1.3	3.5 2.6	4.4

† X-ray illumination limited by beam height to 15mm in EH1 and 28mm in EH2.
